# Early-Warning Assessment of Two-Spot Cotton Leafhopper (*Amrasca biguttula*) Using the Normalized Research Risk Metric (NRRM) Across Multiple Invasive Species

**DOI:** 10.3390/insects17080816

**Published:** 2026-08-06

**Authors:** Muhammad Z. Ahmed, Justin George

**Affiliations:** 1 Pee Dee Research and Education Center, Clemson University, Florence, SC 29506, USA; 2 Southern Insect Management Research Unit, United States Department of Agriculture, Agricultural Research Service, Stoneville, MS 38776, USA

**Keywords:** global research visibility, research momentum, pre-establishment indicators, species emergence patterns, proactive invasion assessment

## Abstract

The two-spot cotton leafhopper (*Amrasca biguttula*) is an emerging invasive pest in the southeastern United States, with recent establishment in cotton, vegetables, and ornamental hibiscus. Its rapid spread, potential economic losses, and regulatory impacts highlight the need for additional early-warning tools capable of identifying high-risk arthropod invaders before widespread establishment. We developed and applied the Normalized Research Risk Metric (NRRM), a literature-based framework that quantifies global research visibility using total publications, recent research, research momentum, and geographical breadth. A comprehensive multi-database Web of Science search was conducted across all available indices using species-specific synonyms, address filters, and harmonized country names. Six globally important invasive species were used to train the model, and *A. biguttula* was evaluated as the primary prediction species. The species exhibited high global research visibility (T = 403; C = 25) and a positive NRRM score (0.651), consistent with its long-recognized pest status in Asia and its rapid emergence in the U.S. In contrast, *Heliococcus summervillei* received a strongly negative NRRM score (−1.14). NRRM provides a rigorous early-warning indicator that can be integrated with ecological and eco-climatic models to further improve the prediction of future invasive threats in agricultural and horticultural systems.

## Simple Summary

The two-spot cotton leafhopper (*Amrasca biguttula*) is a rapidly emerging invasive pest in the southeastern United States. Because it spreads quickly and triggers regulatory actions and economic loss concerns, growers and agencies need tools to identify high-risk invaders before they become widespread. We developed the Normalized Research Risk Metric (NRRM), a literature-based early-warning model that evaluates global research visibility. By applying NRRM to *A. biguttula* and several comparison species, we show that this pest exhibits strong early warning signals consistent with those of major invasive arthropods. NRRM can be used alongside ecological and eco-climatic models to help predict which species are most likely to become future problems in agricultural and ornamental systems. This approach provides a practical, proactive supporting tool for anticipating invasive pests before they cause significant economic damage.

## 1. Introduction

Early assessments of invasive arthropods are often constrained by limited biological, ecological, or distributional data immediately after establishment [1]. This challenge is well documented in invasion biology and affects the reliability of early evaluations [2]. Most early-warning approaches rely on ecological niche models, climate suitability projections, or pathway analyses [1,2,3,4]. These methods require detailed species information that is rarely available during the first months after establishment [1,2,3,4].

Bibliometric indicators provide another source of early information [5,6]. Global publication patterns can reflect long-term research attention, historical pest status, and geographic spread [5,6].

Although bibliometric indicators are widely used to map research fields and identify emerging topics [7,8], they have not been incorporated into an operational invasion-risk framework [9]. A clear methodological gap remains because no published framework uses bibliographic patterns as an early warning tool for invasive arthropods. No standardized approach combines publication volume, research momentum, and geographic breadth into a comparative assessment. We developed the Normalized Research Risk Metric (NRRM) to address this gap. NRRM uses simple bibliometric inputs that can be gathered quickly and applied by users without modeling expertise. It offers early information while ecological, climatic, or biological datasets are still being assembled. NRRM provides an initial signal based on research visibility.

We applied NRRM to the two-spot cotton leafhopper *Amrasca biguttula*, which has recently established in the southeastern United States [10,11]. We compared its profile with the pasture mealybug *Heliococcus summervillei*, which has also established in the region [12]. To train the model, we selected arthropods with different invasion outcomes. The spotted lanternfly *Lycorma delicatula* established and caused major impacts [13]. The armored scale *Fiorinia phantasma* established but spread slowly and caused limited damage [14,15]. Several species are still expanding or emerging as pests, including *Thrips parvispinus* [16,17], *Nipaecoccus viridis* [18,19], *Acanthococcus lagerstroemiae* [20], and *Cydalima perspectalis* [21]. This diversity provides a broad baseline for evaluating early-stage invaders and testing whether NRRM can help distinguish species with higher potential for future impact. Although demonstrated on this set of arthropods, NRRM is designed to support early evaluations by providing a consistent, publication-based indicator that complements existing tools and can be applied broadly wherever publication data are available, especially at early stages with limited technical inputs.

## 2. Materials and Methods

### 2.1. Literature Search and Data Acquisition

All bibliometric data were obtained from the Web of Science platform using the Topic field, which indexes titles, abstracts, author keywords, and Keywords Plus. Searches were conducted on 17 May 2026 across all databases available in Web of Science at the time of extraction. These databases included the Web of Science Core Collection, BIOSIS Citation Index, BIOSIS Previews, Current Contents Connect, Data Citation Index, Derwent Innovations Index, Grants Index, KCI Korean Journal Database, MEDLINE, Policy Citation Index, Preprint Citation Index, ProQuest Dissertations and Theses Citation Index, Research Commons, SciELO Citation Index, and Zoological Record. Species-specific Topic Search strings were used for each taxon. For *Amrasca biguttula*, the search included both “*Amrasca biguttula*” and “*Amrasca devastans*” to capture historical synonymy documented in global phylogeographic analyses. Searches for *Fiorinia phantasma* included “*Fiorinia phantasma*,” “*Fiorinia coronata*,” and “*Fiorinia yongxingensis*” to ensure complete retrieval of adventive and taxonomically related records. For *Heliococcus summervillei*, the search included both “*Heliococcus summervillei*” and its synonym “*Novonilacoccus oryzae*” to capture all published records. For *Acanthococcus lagerstroemiae* and *Cydalima perspectalis*, searches were conducted using only the accepted species names because no additional synonyms were indicated in the available taxonomic sources consulted. All records were exported in text format, and publication years were extracted from the publication year field. The complete global publication dataset used for all analyses is provided in Supplementary File S1.

### 2.2. Dataset Construction and Temporal Alignment

Publication records were grouped by publication year to generate annual global publication counts. Years with no publications were assigned a value of zero. Temporal alignment followed the procedures described by Ahmed and Weeks [22], which ensure that each species’ publication timeline is aligned relative to its U.S. establishment year.

### 2.3. Geographical Dataset and Country Harmonization

After generating global publication counts, a second dataset was created for each species, consisting of records with at least one U.S. author affiliation. These were identified using the Web of Science Address field. For most species, the Address filter AD = “United States” returned complete U.S. subsets. For *A. lagerstroemiae*, *C. perspectalis*, and *H. summervillei*, however, the filter “United States” returned incomplete results, so the alternative filter “USA” was used to obtain full U.S. records. All datasets—global, U.S.-only, and open-access subsets—were processed using identical extraction, grouping, and temporal alignment procedures to maintain comparability across species.

Country names were standardized prior to spatial analysis. Duplicate or overlapping geopolitical labels (e.g., “UK,” “United Kingdom,” “England”; “China,” “Republic of China”) were harmonized following established bibliometric spatial standardization procedures. This ensured that each country contributed a single unique entry to the geographical breadth metric. The cleaned dataset was then used to calculate the number of unique countries contributing at least one publication for each species, providing a measure of global research distribution that has been linked to invasion history and global pest relevance. The harmonized country-level publication dataset used to compute geographical breadth (C) is provided in Supplementary File S2.

### 2.4. Mathematical Structure of the NRRM Model

The Normalized Research Risk Metric is a composite index that integrates three bibliometric predictors of global research visibility. These predictors quantify long-term research attention, recent research acceleration, and global distribution of research activity. The model is designed to provide an early indication of global research visibility prior to U.S. establishment.

#### 2.4.1. Total Global Publications (T)

Total global publications prior to U.S. establishment were calculated as:

$$T=\sum_{y<est}{\mathrm{Publications}}_{y}$$

This metric captures the cumulative global research history of a species. High values of T typically correspond to species with long-recognized pest status or broad economic importance.

#### 2.4.2. Recent Research (R)

Recent research was calculated as the number of publications occurring in the ten years preceding establishment:

$$R=\sum_{est-10\leq y<est}{\mathrm{Publications}}_{y}$$

The ten-year window captures contemporary research momentum while avoiding noise from older literature. This window length is consistent with scientometric analyses of emerging research fronts.

#### 2.4.3. Research Momentum (M)

Research momentum was calculated as the ratio of recent research to total historical research:

$$M=\frac{R}{T}$$

This metric reflects the proportion of recent research relative to total research. High values indicate rapidly increasing research attention, which has been associated with expanding pest ranges and emerging impacts.

#### 2.4.4. Geographical Breadth (C)

Geographical breadth was calculated as:

C=Number of unique countries with at least one publication

This metric reflects the global distribution of research activity. Species with high values of C tend to have broad geographic ranges, multiple invaded regions, or widespread economic importance.

### 2.5. Standardization of Predictors

Because T, M, and C differ in scale and distribution, each predictor was standardized using Z scores. Standardization ensures that each predictor contributes equally to the composite index.

$$Z_{T}=\frac{T-\mu_{T}}{\sigma_{T}}$$

$$Z_{M}=\frac{M-\mu_{M}}{\sigma_{M}}$$

$$Z_{C}=\frac{C-\mu_{C}}{\sigma_{C}}$$

where μ represents the mean of the six training species and σ represents the standard deviation of the six training species.

### 2.6. NRRM Composite Score

The NRRM score was calculated as the arithmetic mean of the three standardized predictors:

$$\mathrm{NRRM}=\frac{Z_{T}+Z_{M}+Z_{C}}{3}$$

Equal weighting is justified because each predictor captures a distinct and non-redundant dimension of research visibility. Training species were assigned NRRM equal to not applicable because they define the standardization baseline.

### 2.7. U.S. Research Response (U)

The U.S. research response was calculated as:

$$U=\sum_{y\geq est}{U.S.\;\mathrm{Publications}}_{y}$$

This metric quantifies the extent of U.S. research activity following establishment. It is not part of the NRRM calculation but provides context for post-establishment research dynamics. The U.S. publication dataset used to compute the U.S. research response (U) is provided in Supplementary File S3.

### 2.8. Data Processing and Visualization

All analyses were conducted in R version 4.4.1. Publication data were imported as tibbles and processed using the tidyverse. Figures were generated using ggplot2 with a minimalist theme. Geographical analyses were conducted in R using the sf package for spatial data handling, rnaturalearth for basemap data, and viridis for perceptually uniform color scaling. Four standalone figures were produced, including global publication trends, research momentum, geographical breadth, and NRRM predictions. All code used for analysis is available upon request.

All analytical code used to compute T, R, M, C, NRRM, and U is provided in Supplementary File S4. All computational procedures described in the Materials and Methods correspond exactly to the analytical workflow implemented in Supplementary File S4. The formulas for T, R, M, C, Z-score standardization, NRRM calculation, and U.S. research response (U) match the code structure precisely. All predictors were computed using the same temporal alignment, species-specific establishment years, and training-species standardization described in the text.

## 3. Results

### 3.1. Global Publication Trends

**Figure 1:**
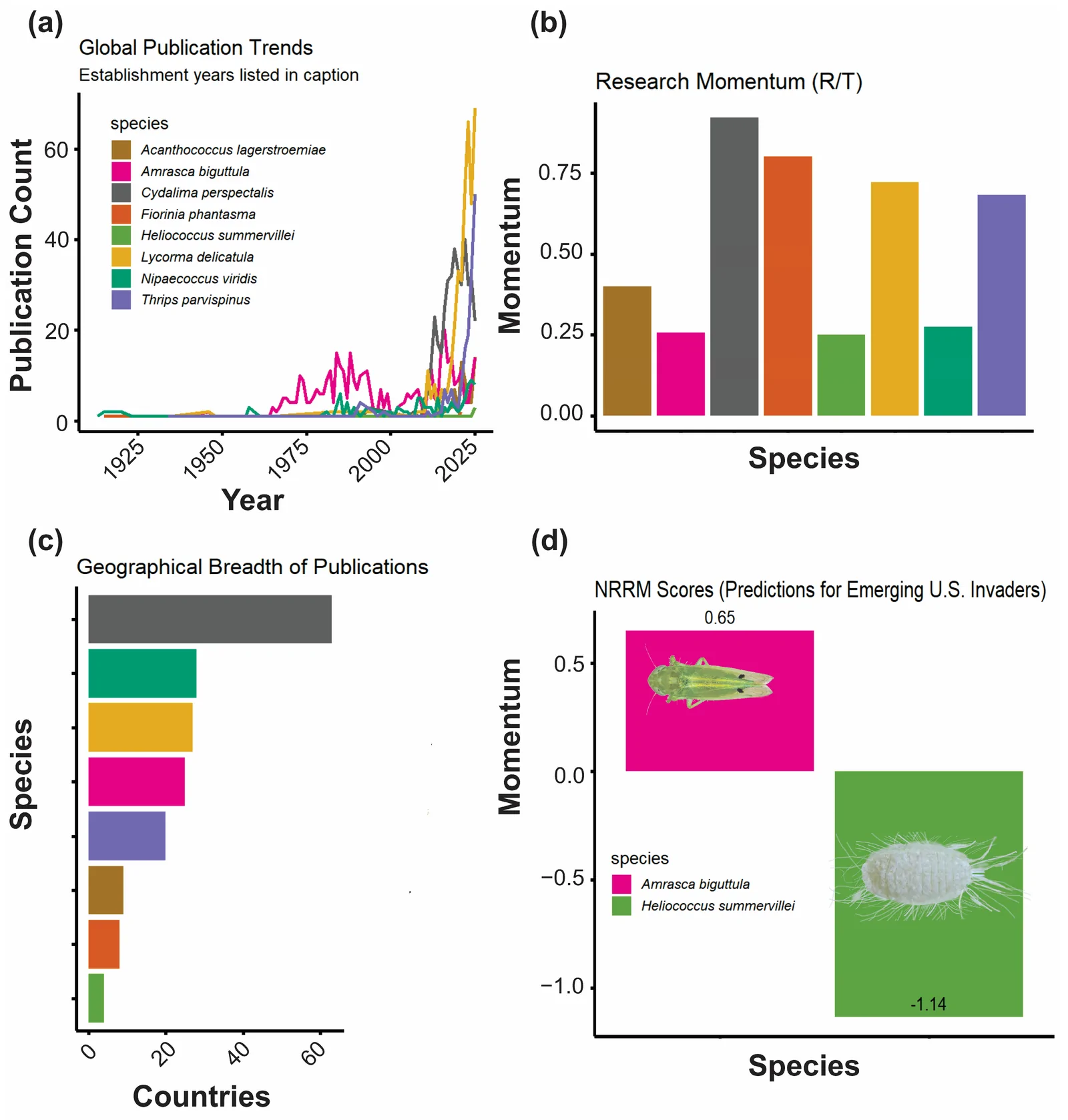
Global research indicators for eight invasive or emerging arthropod pests. (**a**) Global publication trends from the earliest available records through 2025. Establishment years: *Acanthococcus lagerstroemiae* (2004), *Lycorma delicatula* (2014), *Fiorinia phantasma* (2018), *Nipaecoccus viridis* (2020), *Thrips parvispinus* (2020), *Cydalima perspectalis* (2021), *Amrasca biguttula* (2024), and *Heliococcus summervillei* (2025). (**b**) Research momentum (M = R/T), calculated as the proportion of publications occurring in the 10 years preceding U.S. establishment. (**c**) Geographical breadth (C), defined as the number of countries contributing at least one publication for each species. (**d**) NRRM scores for the two prediction species, *Amrasca biguttula* and *Heliococcus summervillei*, based on standardized global research metrics. Positive values indicate higher global research visibility relative to the six training species. *T* refers to total global publications prior to U.S. establishment; *R* represents publications occurring within the 10 years preceding establishment; *M* denotes research momentum calculated as the ratio R/T; *C* indicates geographical breadth, defined as the number of countries contributing at least one publication; and *NRRM* is the Normalized Research Risk Metric derived from standardized values of T, M, and C.

**Figure 2:**
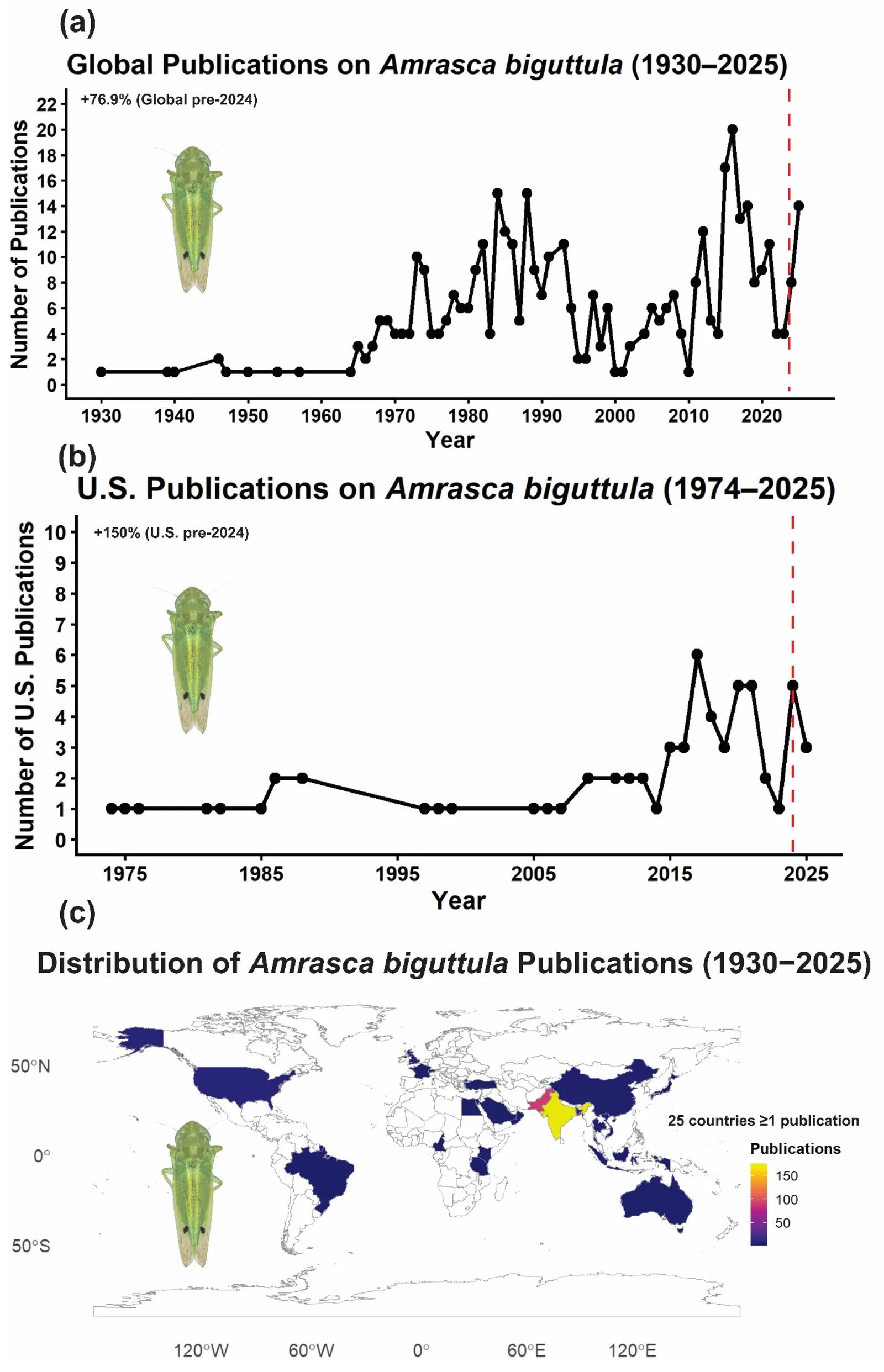
Global, U.S., and geographical publication patterns for *Amrasca biguttula* from 1930 to 2025. (**a**) Global publication trajectory showing a long, continuous research history and a 76.9% increase in mean annual publications between the early historical period (1930–2009) and the recent pre-establishment period (2010–2023). A dashed line marks the 2024 U.S. establishment year. (**b**) U.S. publication trajectory showing low and intermittent research activity prior to establishment, followed by a 150% increase in mean annual publications between the early period (1974–2009) and the recent pre-establishment period (2010–2023). (**c**) Global geographical distribution of publications, showing 25 countries contributing at least one publication. Research activity is strongly concentrated in South Asia, with India and Pakistan accounting for the majority of global output. Countries with no publications are shown in white.

Global publication trajectories varied widely among the eight species examined (Table 1; Figure 1a). *Amrasca biguttula* exhibited the highest total global publication volume prior to U.S. establishment (T = 403), reflecting more than a century of research attention. *Cydalima perspectalis* also showed a substantial global research history (T = 252), with a pronounced increase in publications after 2000. *Nipaecoccus viridis* (T = 102) and *L. delicatula* (T = 43) displayed moderate publication histories consistent with their emergence as high-impact invasive pests. In contrast, *A. lagerstroemiae* (T = 10), *F. phantasma* (T = 5), and *H. summervillei* (T = 4) exhibited sparse and intermittent publication histories, with consistently low annual counts across the entire time series. These patterns are clearly visible in Figure 1a, where species with high T values show long, continuous research trajectories, while species with low T values exhibit minimal or sporadic research activity. Together, species with high total publication counts in Table 1 and Figure 1a represent long-recognized global pests, whereas species with low counts remain comparatively obscure in the global literature prior to their establishment in the U.S. For *A. biguttula*, global publication activity increased steadily over time, with a 76.9% rise in mean annual publications between the early historical period (1930–2009) and the recent pre-establishment period (2010–2023), as shown in Figure 2a.

### 3.2. Research Momentum

Research momentum (M = R/T) differed substantially among species (Table 1; Figure 1b). *Cydalima perspectalis* exhibited the highest momentum (M = 0.921), indicating that most of its research output occurred within the decade preceding establishment. *Fiorinia phantasma* (M = 0.800), *L. delicatula* (M = 0.721), and *T. parvispinus* (M = 0.683) also showed elevated momentum values, reflecting rapid increases in research attention following their emergence as invasive pests. In contrast, *A. biguttula* (M = 0.258) and *N. viridis* (M = 0.275) exhibited low momentum values despite having large or moderate total publication volumes. *Heliococcus summervillei* had the lowest momentum (M = 0.250), consistent with its extremely limited research footprint. These patterns are clearly illustrated in Figure 1b, where species with high momentum cluster near the upper end of the distribution. Together, species with high research momentum in Table 1 and Figure 1b are characteristic of rapidly emerging pests, whereas low momentum reflects either long-established research histories with limited recent activity or minimal global attention.

### 3.3. Geographical Breadth

Geographical breadth (C) ranged from four to sixty-three countries (Table 1; Figure 1c). *Cydalima perspectalis* exhibited the broadest global research distribution (C = 63), followed by *N. viridis* (C = 28), *L. delicatula* (C = 27), and *A. biguttula* (C = 25). *Thrips parvispinus* had moderate breadth (C = 20). In contrast, *A. lagerstroemiae* (C = 9), *F. phantasma* (C = 8), and *H. summervillei* (C = 4) exhibited narrow global research footprints. Figure 1c visually reinforces these differences, with species such as *C. perspectalis* and *N. viridis* occupying the upper range of global distribution, while *H. summervillei* and *F. phantasma* remain at the lower extreme. Together, species with broad geographical research footprints in Table 1 and Figure 1c tend to be globally distributed or widely impactful, whereas species with narrow footprints remain regionally confined or poorly studied.

### 3.4. NRRM Output

The NRRM model produced contrasting scores for the two prediction species (Table 1; Figure 1d). *Amrasca biguttula* received a positive NRRM score of 0.651, driven by a high total publication count (T = 403), moderate geographical breadth (C = 25), and standardized values of Z_T = 3.520, Z_M = −1.520, and Z_C = −0.041. In contrast, *H. summervillei* received a strongly negative NRRM score of −1.140, reflecting extremely low total publications (T = 4), low geographical breadth (C = 4), and low research momentum (M = 0.250), with standardized values of Z_T = −0.768, Z_M = −1.550, and Z_C = −1.090. Figure 1d clearly separates the two prediction species, illustrating the model’s ability to distinguish species with extensive global research visibility from those with minimal global recognition. NRRM values in Table 1 and Figure 1d demonstrate that the model effectively separates globally recognized pests from globally obscure species. For *A. biguttula*, research activity was distributed across 25 countries, with the highest concentration in South Asia, reflecting its long-recognized pest status in the region (Figure 2c).

### 3.5. U.S. Research Response

Post-establishment U.S. research activity varied widely among species (Table 1). *Lycorma delicatula* exhibited the strongest U.S. research response (U = 250), reflecting intense national attention following its rapid spread. *Acanthococcus lagerstroemiae* (U = 46) and *N. viridis* (U = 22) also showed substantial U.S. research activity. In contrast, *A. biguttula* (U = 8), *F. phantasma* (U = 3), *T. parvispinus* (U = 3), and *H. summervillei* (U = 3) exhibited limited U.S. research engagement following establishment. These values indicate that the U.S. research response is strongly influenced by perceived economic impact and rate of spread. U.S. research response values in Table 1 show that species with rapid spread or high economic impact receive the strongest post-establishment attention. U.S. publication activity for *A. biguttula* remained low for several decades but increased by 150% during the recent pre-establishment period (2010–2023), indicating that national research attention began to rise even before the species was detected in 2024 (Figure 2b). Together, the temporal and geographical patterns summarized in Figure 2 illustrate that *A. biguttula* possessed strong global research visibility prior to its U.S. detection, consistent with its long-established importance as a major agricultural pest.

## 4. Discussion

The Normalized Research Risk Metric (NRRM) provided a structured way to compare global research visibility among eight invasive or emerging arthropod pests. NRRM provides early contextual information based on global research visibility. It does not evaluate biological traits, ecological risk, establishment potential, or dispersal capacity. For this reason, NRRM should be interpreted as a complementary bibliometric indicator rather than a predictive invasion risk model. By integrating total global publications, recent research momentum, and geographical breadth, NRRM captured complementary dimensions of research activity that differed substantially among species. These differences reflect long-standing imbalances in global scientific attention, which can influence how quickly a species becomes recognized as a potential threat following introduction [1,2]. For *A. biguttula*, the temporal and geographical patterns shown in Figure 2 reinforce this interpretation: global publication activity increased steadily prior to U.S. detection, and research was distributed across 25 countries, indicating long-standing global visibility before its 2024 establishment. As noted in invasion biology, early assessments often rely on incomplete biological or ecological information [1,2,3,4], and bibliometric indicators can help contextualize species during this information-limited period [5,6,7,8,9,23].

The positive NRRM score for *A. biguttula* was driven by its extensive global publication history and moderate geographical breadth. These values are consistent with its long-established importance as a major agricultural pest in Asia, where it has been the focus of sustained research for many decades [11]. Although its research momentum was relatively low, this pattern is typical of species with long research histories in which publication growth has stabilized. The pre-2024 rise in both global and U.S. publications (Figure 2a,b) further illustrates that research attention was already accelerating before the species was detected domestically. NRRM therefore reflects patterns of research attention rather than ecological impact, and its values should be interpreted as contextual indicators rather than forecasts of establishment or spread [3,4,9,24]. As with any bibliometric approach, Topic searches may retrieve incidental mentions of a species without focusing on it. This limitation is inherent to large literature databases and may introduce noise into publication counts, particularly for species with small research footprints.

In contrast, *H. summervillei* received a strongly negative NRRM score, reflecting extremely low global publication volume, narrow geographical breadth, and low research momentum. These values indicate that the species has attracted minimal global research attention and remains poorly characterized outside of its recent detection in Texas [12]. Despite this limited research footprint, *H. summervillei* is already associated with localized damage, which illustrates that species with low research visibility may still pose risks when ecological conditions are favorable. Similar patterns have been reported for other adventive species that spread cryptically and cause impacts before receiving substantial research attention [5,6]. A low NRRM score, therefore, highlights limited global research rather than low biological risk. These patterns highlight that research visibility does not necessarily correspond to biological risk. Species with low publication volume may still pose ecological or economic threats when environmental conditions are favorable, and NRRM should not be interpreted as evidence of low biological impact.

An additional limitation concerns potential false-negative outcomes for species originating from regions with limited scientific output. Highly invasive taxa may receive low NRRM values simply because they have not yet attracted substantial research attention, which could lead to underestimation of risk if NRRM is interpreted without ecological context. This issue underscores that NRRM reflects research visibility rather than biological threat and should not be used as a standalone indicator for quarantine decision-making. Furthermore, the current implementation assigns equal weight to T, M, and C based on conceptual simplicity rather than statistical optimization, and no assessment of predictor collinearity was performed. Future applications incorporating larger and taxonomically diverse species pools may enable more rigorous approaches—such as principal component analysis, correlation analysis, sensitivity testing of weighting schemes, or regression-based evaluation—to improve model stability, interpretability, and robustness.

Patterns among the six training species further illustrate how NRRM relates to known invasion histories. Species with extensive global spread or high economic relevance, including *C. perspectalis*, *L. delicatula*, and *N. viridis*, exhibited high values for at least one NRRM component. These patterns are consistent with their broad distributions and documented impacts [1,7,13,18,19,20,21]. In contrast, species with limited global research histories, such as *F. phantasma* and *A. lagerstroemiae*, displayed low values across multiple predictors, reflecting their narrower distributions and more recent emergence as pests [14,15,20]. These contrasts show that NRRM distinguishes species with long-standing global visibility from those that remain obscure in the literature. The geographical distribution shown in Figure 2c aligns with these contrasts, as *A. biguttula* exhibits a broader research footprint than several low-visibility species, supporting its placement among globally recognized pests. Because this study represents an initial demonstration of NRRM, the training species set is necessarily limited. The six species were selected to span contrasting invasion trajectories and research profiles, including widely distributed high-impact invaders with extensive research histories, species with moderate or emerging global relevance, and species with narrow distributions and low research visibility. This diversity was chosen to illustrate how NRRM behaves across different invasion contexts in a conceptual demonstration rather than to provide a statistically representative sample. Future applications should incorporate larger and taxonomically stratified species pools to improve the stability, robustness, and generalizability of standardized predictors.

NRRM values were not calculated for the training species because they define the standardization baseline. However, retrospective validation represents a useful direction for future work. For example, reconstructing NRRM values using only pre-establishment data and comparing them with subsequent post-establishment publication trajectories could help evaluate whether high NRRM values correspond to later increases in research attention. Such retrospective tests would strengthen the assessment of NRRM’s predictive utility without altering the current methodological demonstration.

NRRM also provided context for post-establishment research activity, but interpretation depends on the length of the observation window. For species established well before the end of the study period, including *L. delicatula*, *F. phantasma*, *N. viridis*, and *A. lagerstroemiae*, differences in U.S. research response are more informative. *Lycorma delicatula* has attracted substantial national attention, consistent with its rapid spread and high economic concern [13]. *Fiorinia phantasma*, *N. viridis*, and *A. lagerstroemiae* have received more limited but measurable follow-up, reflecting intermediate levels of perceived risk [14,15,18,19,20]. For *A. biguttula* and *H. summervillei*, which were detected only recently in 2024 and 2025 [10,11,12], the short time frame covered by the dataset does not allow a meaningful assessment of U.S. research response. For very recent detections, NRRM is therefore more informative about the global research context than about the national research dynamics.

Although NRRM provides a useful framework for comparing species based on global research patterns, it has important limitations. The model does not incorporate biological traits, climatic suitability, host range, or ecological interactions, all of which influence establishment and spread [3,4,9,24]. NRRM also cannot detect species that have not yet attracted global research attention, as demonstrated by *H. summervillei*. For these reasons, NRRM should be interpreted as a complementary indicator rather than a risk model. Integrating NRRM with ecological niche modeling, climate-based projections, horizon scanning, and field surveillance data will provide a more complete understanding of invasion potential and management priorities [3,4,23,24,25,26].

The current implementation does not include uncertainty analysis, confidence intervals, or error propagation, and the model produces a single composite value without quantifying the effects of parameter changes. This reflects the conceptual nature of the initial demonstration and the limited size of the training set, which restricts the feasibility of formal sensitivity testing. Future applications using larger and more diverse species pools may incorporate bootstrap resampling, cross-validation, or parameter perturbation analyses to evaluate model stability. In addition, all bibliometric data were obtained from Web of Science, which provides harmonized metadata and consistent country-level affiliation fields but may underrepresent regional literature or non-indexed publications. Indexing policies can also influence publication counts. Future work may integrate additional bibliometric sources such as Scopus, Dimensions, or Google Scholar to improve coverage and reduce database-specific bias.

A practical limitation of the current implementation is that NRRM relies on bibliometric data extracted from the Web of Science database, which is subscription-based. Access to Web of Science is typically restricted to universities, research institutes, and government agencies, meaning many frontline plant protection stations, port quarantine units, and small- or medium-sized agricultural operations may lack reliable access to this resource. This constraint limits the operational scalability of NRRM and restricts its direct use by practitioners without institutional database subscriptions. Although the metric itself is computationally simple and low-cost once data are available, the dependence on a paid bibliometric source introduces an accessibility barrier that should be considered when evaluating its practical utility. Future applications may explore open-access bibliometric repositories or streamlined retrieval pipelines to improve accessibility for non-academic users and better align the framework with early-stage needs in field-level plant protection.

A practical advantage of NRRM is that it can be implemented using simple bibliographic queries and basic spreadsheet or scripting tools. The required inputs are publication counts by year and country, which can be obtained from widely used databases without specialized modeling expertise. This makes NRRM accessible to practitioners, students, and agencies that may not have the capacity for complex quantitative modeling but still need a rapid and transparent way to compare research visibility among emerging pests. In this role, NRRM can support early discussions on surveillance priorities, information gaps, and the need for targeted research, particularly when biological and ecological data remain sparse [5,6,7,8,9,23,25,26].

The behavior of the research momentum metric (M) also warrants caution. When total publication counts are very small, M can fluctuate markedly with only a few records, making its interpretation less stable. This sensitivity is expected in early-stage bibliometric datasets and should be considered when comparing species with sparse publication histories. Future applications may also explore alternative formulations or weighting schemes for the momentum component, or minimum publication thresholds, to reduce sensitivity when total publication counts are extremely small.

Overall, NRRM offers a reproducible method for quantifying global research visibility and situating emerging detections within broader research patterns. When interpreted alongside ecological and field-based assessments, it can help identify species that may warrant additional monitoring or research investment without overstating its predictive capacity. The publication patterns summarized in Figure 2 demonstrate how bibliometric signals can reveal emerging relevance well before ecological impacts are documented, underscoring the value of integrating research-visibility metrics into early-stage invasion assessments. For these reasons, NRRM is most informative when interpreted alongside ecological niche models, climate suitability analyses, surveillance data, and expert judgment. NRRM is not intended to replace biological or ecological assessments but to provide an additional literature-based perspective during early information-limited periods.

## 5. Conclusions

The Normalized Research Risk Metric (NRRM) provides a transparent and reproducible framework for quantifying global research visibility for invasive and emerging arthropod pests. By combining total global publications, recent research momentum, and geographical breadth, NRRM summarizes major differences in research attention among species and places new detections into a global context. The contrasting NRRM profiles of *A. biguttula* and *H. summervillei* illustrate how the metric distinguishes species with extensive research histories from those that remain globally obscure [10,11,12]. The temporal and geographical publication patterns summarized in Figure 2 highlight that *A. biguttula* possessed strong global research visibility prior to its U.S. detection, consistent with its long-established importance as a major agricultural pest. Because NRRM does not incorporate biological traits, ecological suitability, establishment likelihood, or potential for spread, it should not be used as a predictive risk model. Instead, it should be interpreted as a contextual bibliometric indicator that summarizes only global research visibility. It offers a rapid and accessible indicator that can be applied by users without modeling expertise, including practitioners and students, wherever publication data are available. As this study represents an initial demonstration, the training species set is necessarily limited. Future applications should incorporate larger and taxonomically diverse species pools to improve standardization and enhance the robustness of NRRM outputs. When interpreted alongside ecological assessments, surveillance data, and expert judgment, NRRM can contribute to early-stage prioritization and help identify species that merit closer monitoring or targeted research [3,4,23,24,25,26].

## Figures and Tables

**Table 1:** NRRM predictor values, standardized scores, and U.S. research response (U).

Species	Est_Year	T	R	M	C	Z_T	Z_M	Z_C	NRRM	U
*Acanthococcus lagerstroemiae*	2004	10	4	0.400	9	−0.703	−0.946	−0.837	NA	46
*Amrasca biguttula*	2024	403	104	0.258	25	3.520	−1.520	−0.041	0.651	8
*Cydalima perspectalis*	2021	252	232	0.921	63	1.900	1.170	1.850	NA	5
*Fiorinia phantasma*	2018	5	4	0.800	8	−0.757	0.677	−0.887	NA	3
*Heliococcus summervillei*	2025	4	1	0.250	4	−0.768	−1.550	−1.090	−1.140	3
*Lycorma delicatula*	2014	43	31	0.721	27	−0.349	0.356	0.058	NA	250
*Nipaecoccus viridis*	2020	102	28	0.275	28	0.285	−1.460	0.108	NA	22
*Thrips parvispinus*	2020	41	28	0.683	20	−0.370	0.202	−0.290	NA	3

T = total global publications prior to the species’ U.S. establishment year.; R = publications in the 10 years preceding establishment; M = research momentum (R/T); C = number of countries with ≥1 publication; Z_T, Z_M, Z_C = standardized values; NRRM = Normalized Research Risk Metric; U = total U.S. publications from the establishment year through 2025 (inclusive). NA = not available.

## Data Availability

The original contributions presented in this study are included in the article/Supplementary Materials. Further inquiries can be directed to the corresponding author.

## Supplementary Materials

The following supporting information can be downloaded at: https://www.mdpi.com/article/10.3390/insects17080816/s1. Supplementary File S1: Global publication dataset used to compute annual publication counts. Supplementary File S2: Harmonized country-level publication dataset used to compute geographical breadth (C). Supplementary File S3: U.S. publication dataset used to compute the U.S. research response (U). Supplementary File S4: R script used to compute T, R, M, C, NRRM, and U, and to generate all figures.
